# 1,2,3-Triazoles and their metal chelates with antimicrobial activity

**DOI:** 10.3389/fchem.2023.1247805

**Published:** 2023-08-10

**Authors:** Lozan Todorov, Irena Kostova

**Affiliations:** Department of Chemistry, Faculty of Pharmacy, Medical University—Sofia, Sofia, Bulgaria

**Keywords:** antimicrobial, transition metal, coordination complex, molecular hybrid, 1,2,3-triazoles

## Abstract

The emergence of drug-resistant bacterial and fungal pathogens has highlighted the urgent need of innovative antimicrobial therapeutics. Transition metal complexes with biologically active ligands (coumarins, terpyridines, triazoles, uracils, etc.) have long been investigated for antimicrobial activity. 1,2,3-Triazoles and their molecular derivatives are well known for a plethora of physiological activities, including antibacterial and antifungal. The aim of the present mini-review is to inform the reader about research conducted on potential antimicrobial 1,2,3-triazole complexes with transition metals. What the authors find surprising is how little such research and experimentation has actually been performed and reported in scientific literature. The goal is to highlight research efforts up to now and impress upon the reader the vast perspectives for novel, effective medicinal substances hidden in this yet unexplored field.

## 1 Introduction

Nitrogen heterocycles have been extensively studied for their overall medicinal properties (Heravi and Zadsirjan, 2020; Kerru et al., 2020) and in particular—antimicrobial activity, both antibacterial and antifungal. 1,2,3-Triazoles, in particular, have gained attention due to their structural versatility and potential for diverse biological activities—antimicrobial (Banu et al., 1999; Yadav et al., 2023), antiviral (Viegas et al., 2020; Chopade and Shaikh, 2022), anticancer (Slavova et al., 2020; Alam, 2022), anti-inflammatory (Kuntala et al., 2021; Matin et al., 2022), central nervous system (Silalai et al., 2022; Khan et al., 2023), anti-SARS-CoV-2 (Hamadi et al., 2022) to name a few. A vast amount of experimentation and literature reports on novel antimicrobial 1,2,3-triazoles has been accumulated over the years and the pace of research efforts seems to only accelerate. For example,’s sake, the authors of this article present several very detailed reviews on antimicrobial triazoles, published just over the past few years (Zhang, 2019; Xu, 2020; Deng et al., 2022; Li and Zhang, 2022; Rammohan et al., 2023). Transition metals have played a part in medicine for millenia now. Gold (Parish, 1992), silver (Barillo and Marx, 2014) and mercury (Norn et al., 2008), for example, have historically been applied as disinfectant, anti-inflammatory and antimicrobial agents. The accidental discovery of cisplatin (Alderden et al., 2006) and its anticancer effect introduced transition metals to modern, science-based medicine. Ruthenium, gold, silver and lanthanum, to name a few (Todorov and Kostova, 2023), followed suit. A number of biological activities of transition metal complexes have been reported over the years.

- Anticancer activity, manifested through apoptosis, tumor growth inhibition and reduction of drug resistance. Different mechanisms of action have been observed -DNA binding and intra-strand adduct formation (Tchounwou et al., 2021) (platinum complexes), DNA groove binding and possible subsequent DNA cleavage (Lee et al., 2020) (ruthenium complexes), interaction of thiol-containing biomolecules like thioredoxin reductase (Zou et al., 2015) (gold complexes), etc;
- Antimicrobial activity against bacteria, fungi and protozoa, including some drug-resistant strains (Frei et al., 2023);
- Antioxidant activity (Rodríguez-Arce and Saldías, 2021; Abd El-Lateef et al., 2023; Todorov et al., 2023), scavenging reactive oxygen species (ROS) thus protecting healthy cells from oxidative stress-related damage;
- Photodynamic therapy (PDT)—some transition metal complexes with organic ligands act as photosensitizers in PDT, generating ROS upon light irradiation (McKenzie et al., 2019). This allows for localized cytotoxicity against cancer cells and pathogenic microorganisms;
- Anti-inflammatory activity (Mucha et al., 2021; Obaleye et al., 2021) by impacting inflammatory pathways (e.g., cyclooxygenase inhibition (Deb et al., 2020)) and suppression of pro-inflammatory molecules (e.g., cytokine suppression (Lin et al., 2022)), showing potential as possible future treatment agents for inflammatory diseases;
- Bioimaging and phototherapeutic agents (Lee and Lo, 2022; Palacio et al., 2022), based on the intrinsic luminescent properties of the transition metals and the “antenna effect” of the coordinated ligands.

Transition metal complexes have been gaining more and more popularity over the past decades as potential antimicrobial agents. Recent increases in the number of drug-resistant pathogens (Waclaw, 2016) have opened a new door for research of transition metal coordination compounds, characterized with novel mechanisms of action and reduced sensitivity to microbial defenses. With that consideration in mind, the authors ventured to review the available scientific literature on the subject of coordination compounds, containing a transition metal ion and 1,2,3-triazole bearing ligands. Considering the large number of 1,2,3-triazoles with reported antimicrobial activity, their complexes with transition metals have sparsely undergone such research. The authors present herewith all currently available data on the subject, revealing a wide field of opportunity for future investigations.

## 2 1,2,3-Triazole complexes with antimicrobial activity

A series of Ni(II) and Co.(II) mixed-ligand complexes with 1,2,3-triazole and thiocyanate were synthesized (Olagboye et al., 2013). These relatively simple structures allow for a more “immediate” observation of the impact of the 1,2,3-triazole ligand on antimicrobial activity. The novel complexes and the respective ligands were tested against bacteria (*Staphylococcus aureus, Escherichia coli, Klebsiella aerogenes, Bacillus subtilis* and *Salmonella typhi*) and fungi (*Aspergillus niger*, *Colletotrichum falcatum*, *Alternaria solani*, *Fusarium oxysporum*, and *Rhizoctonia solani*). Exposure time was 24 h, 37 centigrade, agar pour plate technique at 2.5% and 5.0% concentrations of the tested compounds. Streptomycin sulphate (0.2%) was used as a positive standard for bacteria and benlate—for the fungi. Zone of inhibition radii were used to measure the effect. While all complexes, ligands and simple metal salts were weaker antimicrobials, compared to the positive standards, the authors noted that Co.(II)-1,2,3-triazole complexes were more potent antifungal and antibacterial agents, compared to their Ni(II) counterparts. Coordinating the metal ions with 1,2,3-triazole improves antimicrobial activity, the zone of inhibition of 5.0% CoCl_2_ was 5–12 mm (fungal experiments), coordinating the ion with one molecule 1,2,3-triazole increases the radius to 32–56 mm. 5.0% NiSO_4_ had a zone inhibition radius of 1–16 mm (fungi), coordinating it with one 1,2,3-triazole increases the radius to 61–76 mm. Coordinating a second 1,2,3-triazole causes a further increase to 70–77 mm. Similar observations were made in the bacterial experiments. The aforementioned experiment serves as a simple demonstration how complexation of a transition metal with the 1,2,3-triazole pharmacophore leads to improvement of the biological activity.

Kumar and coworkers (Kumar et al., 2015a) reveals that a Ru(II) triply-stranded helicate complex, involving a pyridyl-1,2,3-triazole ligand and RuCl_3_. The complex was stable over a period of 72 h both in DMSO solution (50 centigrade heating) and in presence of the biological ligand histidine. The ligand itself and the corresponding helicate complex were tested for antimicrobial activity against *Staphylococcus aureus* (Gram-positive) and *Escherichia coli* (Gram-negative), Gentamicin being the positive control. Disk diffusion assays demonstrated that both compounds possess modest activity with Minimum Inhibitory Concentration (MIC) > 256 μg/mL. The authors proposed that dinuclear complexes with increased lipophilicity could improve antibacterial action.

**FIGURE 1:**
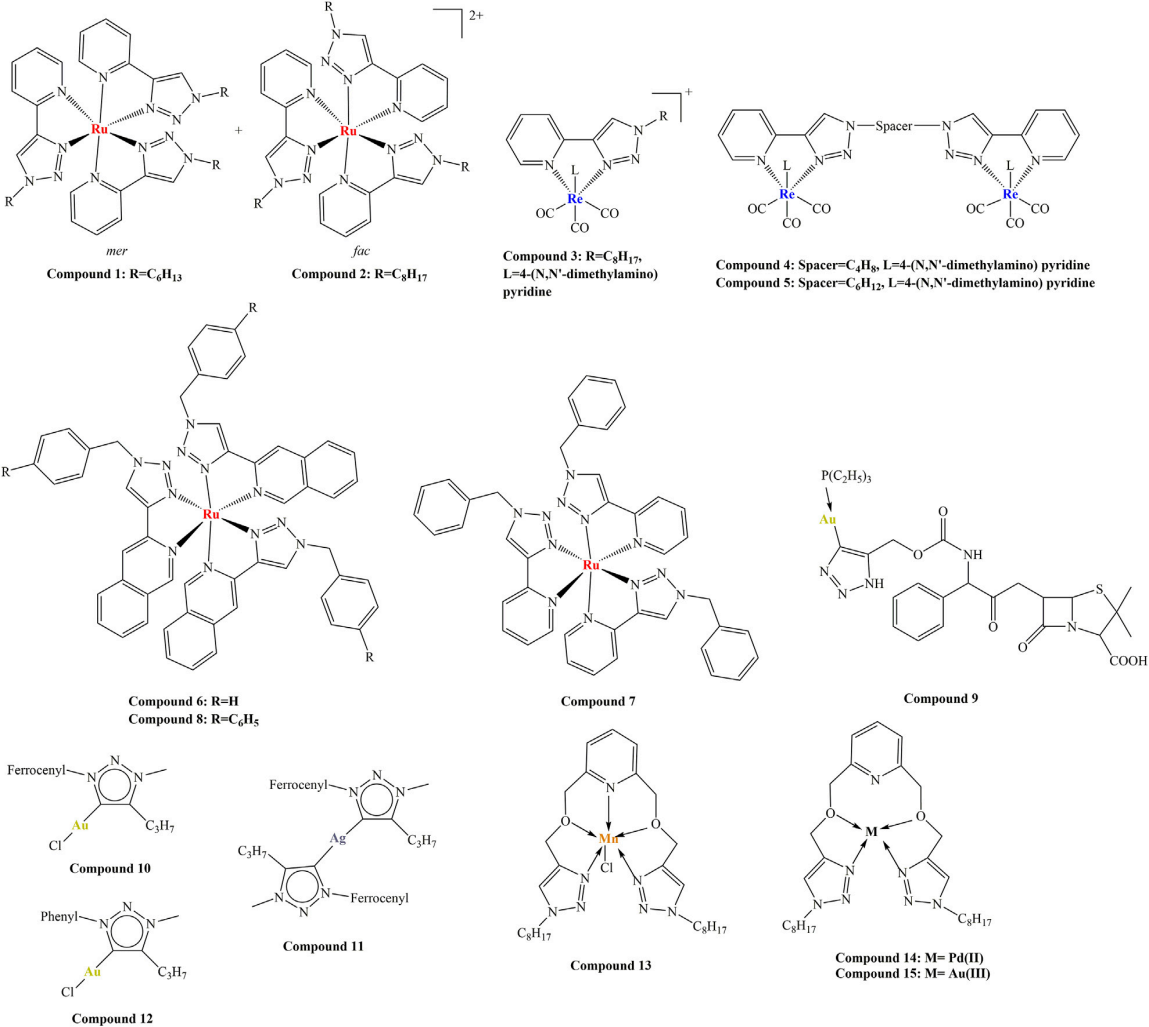
Some of the compounds, reported herein.

Another study involved the synthesis of a series of tris (homoleptic) Ru (II) 2-pyridyl-1,2,3-triazole complexes (Kumar et al., 2016). Ligands were synthesized by way of “click” chemistry and contained various aliphatic butyl, hexyl, octyl, dodecyl, hexadecyl) and aromatic (phenyl, benzyl) moieties. Their antibacterial activities were tested against *S. aureus* (methicillin-resistant (MRSA), strains MR4393 and MR4595 and non-resistant ATCC 25923) and *E. coli* ATCC 25923 by way of disk diffusion, disk dilution and broth microdilution. Gentamicin was used as positive control. Due to limited water solubility, all compounds were tested as DMSO solutions, DMSO having been established to not have biological activity. The most active compounds (Figure 1—compounds 1 and 2) were tested against additional types of pathogenic bacteria—*Acinetobacter calcoaceticus*, and *Mycobacterium smegmatis*. All synthesized ligands did not manifest antimicrobial activity. Complexes bearing an alkyl substituent (6-8 carbon atoms length) at the triazole ring manifested significant antimicrobial activity against non-resistant gram-positive *S. aureus* with MIC between 4 and 8 μg/mL. Promising MICs (4–16 μg/mL) were observed in the resistant *S. aureus* strains, the same or an improved value, compared to Gentamicin (MIC = 16 μg/mL against these same strains). All complexes did not perform well against gram-positive *E. coli*, no effect having been observed within tested concentration ranges. Longer side chains or aromatic substituents were associated with moderate activity. Increased lipophilicity was discovered to be associated with an improved effect, however, the correlation was observed up to a point. The type of geometric isomer (*mer-*or *fac-*) did not impact biological activity. The main mode of action was suggested to be cell wall/cell membrane disruption (1 h incubation in presence of varying concentrations of the complexes, together with fluorescent propidium iodide, followed by fluorescence measurement). The authors dismissed the redox properties of the Ru (II) ion to be involved in the observed biological activity. Cytotoxicity against healthy human dermal keratinocytes and Vero cells showed cytotoxicity well above MIC against the pathogenic bacteria tested. It was noted that stereochemistry did not significantly impact antimicrobial properties.

Similar mono- and di-*fac*-rhenium (I) tricarbonyl 2-pyridyl-1,2,3-triazole complexes with a variety of aliphatic and aromatic substituents were synthesized (Kumar et al., 2015b). None of the ligands exhibited antibacterial activity *versus E. coli* (ATCC 25922) and *S. aureus* (ATCC 25923). None of the mono-*fac*-tricarbonyl complexes, neutral and cationic, exhibited any activity against Gram-negative *E. coli*. Only one of them exhibited significant activity against *S. aureus* with MIC = 16 μg/mL (Figure 1, compound 3). The positive control, Gentamicin, had MIC< 0.5 μg/mL. The cationic and neutral di-*fac*-tricarbonyl complexes behaved in a similar manner. Only two of them (Figure 1, compounds 4 and 5) exhibited MIC = 16 μg/mL against *S. aureus*, significantly greater than that of Gentamicin. All but two were inactive against *E. coli*. Both active complexes manifested greater activity against *E. coli* MIC = 9 and 15 μg/mL, compared to the positive control Gentamicin MIC = 23 μg/mL. The highest activity is observed in cationic di-rhenium complexes with greater lipophilicity. The authors noted that their Ru (II) analogues are much more active, thus more appropriate as potential antibacterial agents.

Kreofsky and coworkers generated series of Ru(II) coordination compounds of N-N bidentate chelators with 1,2,3-triazole and isoquinoline subunits (Kreofsky et al., 2020): 1-(1-substituted-1,2,3-triazol-4-yl) isoquinolines and 3-(1-substituted-1,2,3-triazol-4-yl) isoquinolines. Each chelator formed 3:1 coordination compound with Ru (II) as a mixture of stereoisomers with the common formula [RuL_3_]Cl_2_. The antimicrobial properties of these compounds were tested against Gram-positive *Bacillus subtilis* and *Staphylococcus epidermidis* as well as Gram-negative *Escherichia coli* and *Enterobacter aerogenes*. None of the ligands manifested inhibition at concentrations up to 250 uM. Coordinating them with Ru (II) caused dramatic improvement in antimicrobial activity. Isoquinoline stereochemistry did not impact antimicrobial properties. Incorporation of isoquinoline (Figure 1, compound 6), instead of pyridine (Figure 1, compound 7) improved activity 3-6 fold. In benzyl-containing (position 1 of the 1,2,3-triazole ring) compounds, isoquinoline groups improved potency against both gram-positive (20 to 40-fold) and gram-negative (8-fold) bacteria. Improving hydrophobicity by way of phenylbenzyl groups (Figure 1, compound 8) instead of benzyl groups (Figure 1, compound 6) caused a diminishment of activity (15-fold against all tested microorganisms). Replacement of the pyridine N-N chelator unit with isoquinoline caused up to 32-fold improvement in MIC values against both Gram-positive (MIC decreased from 16 μM to 0.4–0.8 μM) and Gram-negative bacteria (MIC decreased from more than 250 μM–31 μM). Gram-positive microorganisms were more sensitive to the Ru(II) complexes, compared to the Gram-negative ones. The eukaryotic C. albicans manifested very high MIC values (more than 250 μM with all tested compounds), demonstrating good selectivity against prokaryotes.

Michaut and coworkers synthesized organogold(I) antibacterial compounds by click chemistry with triethylphosphine-gold(I) azides and an alkyne derivative. They reported a novel series of metallo-antibiotics (chryso-lactams), containing penam-scaffolds, a 1,2,3-triazole and a gold(I) ion (Michaut et al., 2020). The novel compounds were tested against a range of Gram-positive (*Staphylococcus aureus, Staphylococcus epidermis, Enterococcus faecalis and E. faecium*) and one Gram-negative (*Escherichia coli*) bacteria. *E. coli* turned out to be resistant to the novel metallo-antibiotics, due to the low permeability of its outer membrane. All novel compounds were more active than their organic, antibiotic precursors. The size of the phosphine ligand of the gold (I) coordination center seemed to significantly impact biological activity. Triphenylphosphine resulted in lower activity, compared to ampicillin. Dimethylphosphine, a ligand with a significantly decreased size, improved activity, compared to triphenylphosphine. Trimethylphosphine did not further improve activity against Gram-positive bacteria, but caused moderate anti *E. coli* activity. The research team noted that smaller phosphine ligands tended to improve activity. The most active compound, (Figure 1, compound 9), bearing a triethylphposphine, was more effective than ampicillin against *Staphylococcus* species and *Enterococcus* strains. It exhibited MIC values of less than 0.05 μg/mL up to 1 μg/mL in the tested Gram-positive strains. Its toxicity against normal human hepatocytes was presented as EC_50_ = 16.39 μg/mL and at concentrations of up to 7.9 μg/mL cell viability was unaffected. Further study by the same team (Michaut et al., 2021) on the impact of the penam scaffold on antibacterial activity against the same bacterial strains showed that chirality of the phenyl-glycine core appeared to have no effect. The penam scaffold was dispensable, according to the authors, since antibacterial activity of the investigated substances relied on the gold (I) ion, thus reducing the risk of emergence of cross-resistance with other antibiotic families.

Hoyer and coworkers (Hoyer et al., 2021) synthesized series of silver (I) and gold (I) complexes with mesoionic carbenes of 1,2,3-triazole-5-ylidene type. Antibacterial activity was investigated with Gram-negative *Salmonella typhimurium* (liquid microdilution assay) and *Escherichia coli*. Initial steps of the experiment involved the synthesis of triazoles and triazolium salts that bore no significant antibacterial activity. Contrary to previous observations, addition of a ferrocene functionality to position 1 of the triazole ring (Figure 1, compounds 10 and 11) decreased the antibacterial effect, compared to a phenyl functionality in the same position (Figure 1, compound 12). Complexation with Ag (I) and Au (I) improved activity, the silver complexes being more active than the gold ones.

Abdulameer et al synthesized complexes of Mn (II), Pd (II) and Au (III) with 6-bis (((1-octyl-1H-1,2,3-triazol-4-yl) methoxy) methyl) pyridine (Abdulameer and Alias, 2022). As a result of rigorous analysis, involving a variety of analytical methods, the authors concluded that the ligand can behave as a tetradentate and pentadentate chelator. The Mn complex had octahedral geometry, while the Pd and Au had square planarl geometry (Figure 1, compounds 13–15). They were tested for antimicrobial activity against *S. aureus, E. coli* and *C. albicans.* Their potency was measured as width of the inhibition zone, associated with the compound’s presence at three different concentrations (10, 50, and 200 ppm). At all investigated concentrations and with all microbes tested, the observed activity increased in the following order: ligand < Mn complex < Pd complex < Au complex. The most potent compound, the Au (III) complex, at the highest dose of 200 ppm manifested inhibition diameters of 35 mm (*E. coli*), 26 mm (*S. aureus*) and 18 mm (*C. albicans*). The researchers did not provide results from a positive control in their article.

Aljohani et al. (2022) and coworkers synthesized 1,2,3-triazole-sulfadiazine-ZnO hybrids and tested them against three carbapenem-resistant metallo-β-lactamase producing *K. pneumoniae* (Kp1, Kp5 and Kp8). The synthesized novel sulfadiazines exhibited MIC values between 128 and 256 μg/mL against all three strains. The ZnO nanoparticles, used in the hybrids, had MIC between 16 and 32 μg/mL. Combining the novel sulfadiazines with the ZnO nanoparticles caused a significant synergistic effect (MIC between 2 and 18 μg/mL). Cell viability assays with normal lung cells (CEAS-2Bs) showed IC_50_ values between 160 and 620 μg/mL. These results demonstrated potential excellent activity *versus* resistant microbial strains, combined with comparatively low toxicity against healthy tissues. Endotracheal aerosolization of the most potent substance revealed its presence in rat’s lungs after 24 h (residence duration up to 85.3%). That is combined with notable ability to control pneumonia infection in rats, revealed by histopathological investigations.

Smitten and coworkers synthesized 1,2,3-triazole-based Os(II) complex as prospective cellular imaging agents, testing their red/near-IR luminescence and antimicrobial activity (Smitten et al., 2020). The novel, water-soluble complexes were tested against a panel or resistant cell lines—methicillin resistant *Staphylococcus aureus* (MRSA), uropathogenic, multidrug resistant EC958 ST131 strain of *Escherichia coli*, a multidrug resistant clinical isolate strain of *Pseudomonas aeruginosa*—PA2017 and a multidrug resistant clinical isolate strain of *Acinetobacter baumannii*—AB184. MICs were obtained in nutrient rich media Mueller–Hinton-II (MH-II) and defined media—Glucose Defined Minimal Media (GDMM) for Gram-negative strains and Chemical Defined Media (CDM) for Gram-positive strains. All complexes manifested no activity (same as the positive control Ampicillin) against almost all pathogenic strains. The only exception being MRSA, with which some mild activity was observed (MIC = 48 μg/mL and 96 μg/mL) by two of the complexes. It was noted that *mer*-complexes possessed higher antibacterial effect than their *fac*-isomers. Potential phototoxicity was also investigated. The researchers discovered that light irradiation (48 J/cm^-2^, 30 min) does improve antibacterial activity compared to the control in dark conditions.

A summary of the data on antimicrobial activities of the most active compounds, described herein, can be viewed in Table 1.

**TABLE 1:** Antimicrobial activities of the compounds, described herein, that manifest significant antimicrobial activity.

Compound number	Microbial strain	Activity—tested compound	Activity—control substance (if tested)
1	*S. aureus* (ATCC 25923)	MIC	Gentamicin (MIC)
		8 μg/mL	< 0.125 μg/mL
	*S. aureus* (NZRM 9653)	1 μg/mL	0.5 μg/mL
	*MRSA* (MR 9519)	4 μg/mL	0.125 μg/mL
	*MRSA* (MR 4393)	4 μg/mL	16 μg/mL
	*MRSA* (MR 4549)	8 μg/mL	16 μg/mL
	*M. smegmatis*	4 μg/mL	0.125 μg/mL
	*A. calcoaceticus*	128 μg/mL	64 μg/mL
2	*S. aureus* (ATCC 25923)	MIC	Gentamicin (MIC)
		8 μg/mL	< 0.125 μg/mL
	*S. aureus* (NZRM 9653)	2 μg/mL	0.5 μg/mL
	*MRSA* (MR 9519)	8 μg/mL	0.125 μg/mL
	*MRSA* (MR 4393)	4 μg/mL	16 μg/mL
	*MRSA* (MR 4549)	8 μg/mL	16 μg/mL
	*M. smegmatis*	8 μg/mL	0.125 μg/mL
	*A. calcoaceticus*	16 μg/mL	64 μg/mL
3	*S. aureus* (ATCC 25923)	MIC	Gentamicin (MIC)
		16 μg/mL	< 0.5 μg/mL
	*E. coli* (ATCC 25922)	none	< 0.5 μg/mL
4	*S. aureus* (ATCC 25923)	MIC	Gentamicin (MIC)
		16 μg/mL	< 0.5 μg/mL
	*E. coli* (ATCC 25922)	none	< 0.5 μg/mL
5	*S. aureus* (ATCC 25923)	MIC	Gentamicin (MIC)
		32 μg/mL	< 0.5 μg/mL
	*E. coli* (ATCC 25922)	none	< 0.5 μg/mL
6	*B. subtilis* (ATCC 6051)	MIC	No control tested
		0.8 μM	
	*S. epidermidis* (ATCC 14990)	0.4 μM	
	*E. coli* (ATCC 25922)	31 μM	
	*E. aerogenes* (ATCC 13048)	31 μM	
	*C. albicans* (ATCC 90028)	250 μM	
7	*B. subtilis* (ATCC 6051)	MIC	No control tested
		>250 μM	
	*S. epidermidis* (ATCC 14990)	>250 μM	
	*E. coli* (ATCC 25922)	>250 μM	
	*E. aerogenes* (ATCC 13048)	>250 μM	
	*C. albicans* (ATCC 90028)	>250 μM	
8	*B. subtilis* (ATCC 6051)	MIC	No control tested
		16 μM	
	*S. epidermidis* (ATCC 14990)	16 μM	
	*E. coli* (ATCC 25922)	>250 μM	
	*E. aerogenes* (ATCC 13048)	>250 μM	
	*C. albicans* (ATCC 90028)	>250 μM	
9	*S. aureus* (ATCC 25923)	MIC	Ampicillin (MIC)
		0.125 μg/mL	0.5 μg/mL
	*S. aureus* (ATCC 700699)	0.25 μg/mL	>8 μg/mL
	*S. aureus* (ATCC 29213)	0.125 μg/mL	0.5 μg/mL
	*S. aureus* (ST20131,365)	0.25 μg/mL	4 μg/mL
	*S. epidermidis* (ATCC 14990)	≤ 0.06 μg/mL	1 μg/mL
	*S. epidermidis* (ATCC 35984)	≤ 0.06 μg/mL	>8 μg/mL
	*S. epidermidis* (ST20140436)	≤ 0.06 μg/mL	4 μg/mL
	*S. epidermidis* (ST20150446)	0.125 μg/mL	8 μg/mL
	*E. faecalis* (JH2-2)	0.5 μg/mL	0.5 μg/mL
	*E. faecalis* (UCN41)	1 μg/mL	0.5 μg/mL
	*E. faecalis* (V583)	1 μg/mL	0.5 μg/mL
	*E. faecium* (ATCC 19434T)	1 μg/mL	0.5 μg/mL
	*E. faecium* (BM 4147)	2 μg/mL	8 μg/mL
	*E. faecium* (AUS0004)	1 μg/mL	>8 μg/mL
	*E. coli* (ATCC 25922)	>8 μg/mL	2 μg/mL
10	*S. typhimurium* (strain not indicated)	Relative growth^1^	No control tested
		≈0.4 a.u	
11	*S. typhimurium* (strain not indicated)	Relative growth^1^	Kanamycin (OD_600_^2^, 50 μM)
		≈0.15 a.u	
		OD_600_^2^ at 50 μM	≈0
		≈0.35	
12	*S. typhimurium* (strain not indicated)	Relative growth^1^	Kanamycin (OD_600_^2^, 50 μM)
		< 0.1 a.u	
		OD_600_^2^ at 50 μM	≈0
		≈0.30	
13	*E. coli* (strain not indicated)	Growth inhibition^3^	No control tested
		25 mm	
	*S. aureus* (strain not indicated)	22 mm	
	*C. albicans* (strain not indicated)	16 mm	
14	*E. coli* (strain not indicated)	Growth inhibition^3^	No control tested
		32 mm	
	*S. aureus* (strain not indicated)	24 mm	
	*C. albicans* (strain not indicated)	17 mm	
15	*E. coli* (strain not indicated)	Growth inhibition^3^	No control tested
		35 mm	
	*S. aureus* (strain not indicated)	26 mm	
	*C. albicans* (strain not indicated)	18 mm	

^1^Relative growth was described by the original authors as follows.

*Relative growth= OD*_*600*_ (*bacterium + compound*)*—OD*_*600*_ (*compound*)*,* where OD_600_ is the optical density of the sample at 600 nm. Results are presented for 4 μg/mL. No MICs, were presented.

^2^OD_600_ was used by the original authors as a signal for biological activity when comparing compounds 11 and 12 to Kanamycin. No such comparison was performed for Compound 10. No MICs, were presented. Results shown are for 50 μM concentration.

^3^Presented as width of the inhibition zone in millimeters. Results presented are for the highest concentration tested (200 ppm).

## 3 Discussion and conclusion

From the published material the authors were able to gather, several tentative conclusions could be derived.

- Combining transition metal ions with low, or even nonexistent, antimicrobial activity with otherwise inactive 1,2,3-triazoles can produce active complexes. Such effects of complexation have previously been observed in transition metal complexes with anticancer properties (Todorov et al., 2023);
- In all works reported herein, synergistic effects between the ligand and the metal ion/nanoparticle have been observed;
- 1,2,3-Triazoles can be utilized as molecular “bridges”, connecting transition metal ions to biologically active molecules (e.g., antibiotics, antifungal). This could yield novel antimicrobial agents, with new mechanisms of action, associated with the metal ion, thus overcoming microbial resistance mechanisms;
- Gram-positive bacteria seem to be more sensitive to transition metal complexes, compared to gram-negative. Lipophilicity of the complexes seems to play an important role for the antibacterial effect. There seems to be some kind of a lipophilic “goldilocks zone”, where effectiveness is optimal;
- Results reported herein demonstrate the possibility of creating selective antibacterial agents, that are well tolerated by healthy human tissues, i.e., medicinal substances with favorable therapeutic indices.

The authors of the present article would like to emphasize the gap in research when it comes to antimicrobial transition metal complexes with 1,2,3-triazoles. Given the extensive investigational work that has been done in the field of 1,2,3-triazole molecules/molecular hybrids with antibacterial and antifungal properties, it is highly unusual that their coordination compounds have been somewhat “left behind” in that respect until now. One possible reason might be that most investigations, associated with these types of compounds, are directed at cancer research, with antibacterial/antifungal assays being left as somewhat of an afterthought. The ever-increasing number of multidrug-resistant pathogens, resulting from the ubiquitous application of antimicrobial agents in the fields of human medicine, animal husbandry and agriculture, presents a clear and present threat to human society. The aforementioned research gap could provide a perspective path to the discovery of novel, antimicrobial medicinal compounds that are effective against what the general public might call “superbugs”. It is the authors’ opinion that significantly more research into this matter would be beneficial to all. Synthesis of novel 1,2,3-triazole complexes with platinum, ruthenium, gold and their subsequent testing not only for anticancer, but also for antimicrobial properties could be carried out. In addition, certain other transition metals, like members of the lanthanide series, are known to suppress bacteria (Balusamy et al., 2012). Such metals that are characterized by comparatively low toxicities (Kajjumba et al., 2021) and are relatively abundant in nature which makes them good candidates for novel antimicrobial agents.
